# Cdk5-mediated Drp1 phosphorylation drives mitochondrial defects and neuronal apoptosis in radiation-induced optic neuropathy

**DOI:** 10.1038/s41419-020-02922-y

**Published:** 2020-09-03

**Authors:** Rong Rong, Xiaobo Xia, Haiqin Peng, Haibo Li, Mengling You, Zhuotao Liang, Fei Yao, Xueyan Yao, Kun Xiong, Jufang Huang, Rongrong Zhou, Dan Ji

**Affiliations:** 1 Eye Center of Xiangya Hospital, Central South University, 410008 Changsha, Hunan P.R. China; 2 Hunan Key Laboratory of Ophthalmology, 410008 Changsha, Hunan P.R. China; 3 Department of Oncology, Xiangya Hospital, Central South University, 410008 Changsha, Hunan P.R. China; 4 Department of Spine Surgery, Xiangya Hospital, Central South University, 410008 Changsha, Hunan P.R. China; 5 Department of Anatomy and Neurobiology, School of Basic Medical Sciences, Central South University, 410008 Changsha, Hunan P.R. China

**Keywords:** Molecular neuroscience, Neurological disorders

## Abstract

Radiation-induced optic neuropathy (RION) is a devastating complication following external beam radiation therapy (EBRT) that leads to acute vision loss. To date, no efficient, available treatment for this complication, due partly to the lack of understanding regarding the developmental processes behind RION. Here, we report radiation caused changes in mitochondrial dynamics by regulating the mitochondrial fission proteins dynamin-related protein 1 (Drp1) and fission-1 (Fis1). Concurrent with an excessive production of reactive oxygen species (ROS), both neuronal injury and visual dysfunction resulted. Further, our findings delineate an important mechanism by which cyclin-dependent kinase 5 (Cdk5)-mediated phosphorylation of Drp1 (Ser616) regulates defects in mitochondrial dynamics associated with neuronal injury in the development of RION. Both the pharmacological inhibition of Cdk5 by roscovitine and the inhibition of Drp1 by mdivi-1 inhibited mitochondrial fission and the production of ROS associated with radiation-induced neuronal loss. Taken together, these findings may have clinical significance in preventing the development of RION.

## Introduction

Radiotherapy is currently one of the primary treatments for head, neck, and/or skull-based tumors. Despite its prevalence, this treatment approach is plagued by serious complications^1^. Of these, radiation-induced optic neuropathy (RION) is a severe, late-onset complication caused by radiation therapy (RT)^2^. RION may occur either unilaterally or bilaterally and lead to sudden or progressive irreversible visual field defects and loss of vision^3^. At present, the detailed pathogenesis of RION remains unclear, but involves damage to the optic nerve, resulting in injury of retinal ganglion cells (RGCs) and advancement of ischemic necrosis^4,5^. An in-depth investigation into the molecular pathogenesis of RION would help us to identify efficacious interventions for stabilizing and/or reversing RION-mediated visual decline, ultimately improving the quality of life of cancer patients receiving radiotherapy.

Past work has shown that mitochondria are highly sensitive to ionizing radiation, which induces changes in mitochondrial dynamics, eventually resulting in mitochondrial damage^6^. Mitochondrial dynamics refers to the regulation of mitochondrial morphology, number, and distribution as well as mitochondrial axonal transport by means of continuous fusion and division. For instance, Kempf et al. evaluated the proteomics and transcriptome in the hippocampus and cerebral cortical neurons of mice exposed to X-ray (0.1 and 0.5 Gy). They found that signal pathways involved in mitochondrial function and neuronal synapsis changed, which caused decreases and alterations in the function of neuronal mitochondria, axons, and dendrites^7^. Interestingly, Yamamori et al. used mouse embryonic fibroblast (MEF) cells exposed to 10 Gy of X-ray and showed that mitochondrial homeostasis was essential for X-ray-induced mitotic catastrophe (MC), mitochondrial membrane potential devastation, and cell apoptosis. X-ray exposure increased the expression of dynamin-related protein 1 (Drp1) in MEF cells and induced increased mitochondrial fragmentation. Either inhibiting Drp1 expression or interfering its function could reduce mitochondrial fragmentation and MC, thus promoting cellular survival^8^. In addition, the activity of Drp1 in regulating mitochondrial division is controlled by post-translational modifications to the Drp1 variable domain, including phosphorylation, ubiquitination, and others. Drp1 phosphorylation is regulated by a variety of kinases. For example, serine 616 (Ser616) is a Drp1 phosphorylation site that can be phosphorylated by cyclin-dependent kinase 1 (Cdk1), calmodulin-dependent protein kinase II (CaMKII), and others^6,9,10^. In particular, Ser616 phosphorylation has been shown to cause excessive mitochondrial division, which may result in cardiocyte and hepatocyte injury^10,11^. In contrast, Drp1 phosphorylation at serine 637 (Ser637) is regulated by protein kinase A (PKA), which exerts an inhibitory effect on mitochondrial transport and promotes mitochondrial fusion^12^. Additionally, the large number of reactive oxygen species (ROS) that are overproduced after radiation are present both inside and outside the cell and can either directly or indirectly cause DNA damage, mitochondrial abnormalities, and/or apoptosis^13,14^.

Our study established a causal role of Drp1-mediated mitochondrial kinetic abnormalities, and ROS overproduction in radiation-induced optic neuropathy. Moreover, we also explored the potential therapeutic effect of two inhibitors by alleviating and/or limiting RION progression related to the Cdk5/Drp1 pathway. Collectively, our results may help determine the specific role of both Cdk5 and Drp1 in RION pathogenesis. In turn, this understanding could provide a solid theoretical and practical foundation for use in the development of targeted therapies for RION.

## Materials and methods

### Cell culture

R28 cells, a retinal precursor cell line and have the potential for differentiations, could express specific antigen Thy 1.1 of RGCs^15^, and are commonly used to study neuronal function and neuroprotection in vitro^16^, which were obtained from the Department of Anatomy and Neurobiology of Central South University (Changsha, China). Cells were expanded as previously reported^17^ and cultured in low glucose Dulbecco’s modified Eagle’s medium (DMEM; Hyclone, UT, USA) supplemented with 10% fetal calf serum (Gibco, Carlsbad, CA) and 1% penicillin-streptomycin (Yeasen, Shanghai, China). Cells were maintained in a humidified incubator at 37 °C with an atmosphere containing 5% CO_2_. The cells were regularly tested for mycoplasma contamination using One-step Quickcolor Mycoplasma Detection Kit according to the manufacturer’s instructions (Shanghai, China).

### Animals

Seven-week-old (20–25 g), specific pathogen free (SPF), male BALB/c mice were used in this study. Mice were housed in separate cages at the Central South University Animal Department (Changsha, China) under controlled temperature (21 ± 1 °C), humidity (55 ± 5%), and light/dark cycles (12/12 h). Food and water were available ad libitum. All animals were treated according to the Association for Research in Vision and Ophthalmology Resolution on the Use of Animals in Research. All animal experiments were reviewed and approved by the Animal Care and Use Committees of the Laboratory Animal Research Center at Xiangya Medical School of Central South University (approval ID: SYXK 2015–0017).

### Irradiation

R28 cells were X-ray-exposed at room temperature and irradiated with 0 (sham treatment), 2, 6, or 10 Gy X-rays at a dose rate of 600 rad/min using a Varian Truebeam Linear Accelerator (Varian Clinac 23EX, California, US). Once irradiated, the cells were returned to the incubator until the designated harvest time. Animals were anesthetized with 1% pentobarbital sodium by intraperitoneal injection and then irradiated with 0 (sham treatment), 8, 10, or 12 Gy X-rays at a dose rate of 600 rad/min. Only the heads of the mice were exposed to the radiation field. Once irradiated, the mice were returned to the vivarium until handling at the designated time. Mdivi-1 (M0199) and Roscovitine (R7772) were both purchased from Sigma–Aldrich (St Louis, MO). R28 cells were treated with drug 2 h prior to irradiation and incubated for the indicated times. For animal experiments, Mdivi-1 and Roscovitine were administered using intraperitoneal (i.p.) injection of either 3 mg/kg or 3.5 mg/kg, respectively. Drugs were injected 6 h before irradiation; following, mice received a daily injection for 1 week after irradiation. Doses were selected based on previously reported work^18–21^. Dimethyl sulfoxide (DMSO; Sigma–Aldrich) was used as the solvent for drug administration; no more than 0.1% was used in each experiment.

### Cell viability assay

Cell viability was evaluated using a commercially available Cell Counting Kit-8 (CCK-8) viability assay kit (Yeasen) by following the manufacturer’s instructions. Absorbance was measured at 450 nm using an Infinite M200 Microplate Reader (Tecan, Männedorf, CH).

### Visual-evoked potential (VEP) examination

We recorded flash visual-evoked potentials (fVEPs) using a Flash Visual Evoked Potential Testing System Device (GOTEC Medical, Chongqing, China) as previously described^22^. Briefly, mice were anesthetized with pentobarbital sodium, and lidocaine hydrochloride was applied as a corneal anesthesia. Ocular hydration was maintained using 0.1% sodium hyaluronate eye drops. Animals were placed on a thermoregulated heating pad and secured using a tooth-holder headset. Three silver plate electrodes were separately inserted under the skin of the occipital bone (anode), anterior bregma (cathode), and ear (ground electrode). Unilateral fVEP was recorded with the right eye and left eye covered using foil paper, then exchanged. A short resting period of 5 min was instituted before and between each recording, during which time the room was darkened. The recordings were obtained under very dim room light with a stimuli light of 10.0 cd·s/m2 flash intensity, 1 HZ flash frequency, and 64 times flash. After testing, the animals received topical antibiotics and were allowed to recover in a 37 °C warming chamber. Once ambulatory, mice were returned to their cages. Three distinct amplitudes and latencies from the characteristic fVEP waveform were manually quantified in a blinded fashion for each eye of all mice.

### ROS measurement

The cell permeable reagent 2′,7′-dichlorofluorescein diacetate (DCFH-DA; Sigma–Aldrich) was used to detect post-irradiation ROS production in both R28 cells and mouse retinas, and MitoSOX Red (Yeasen) was used to detect mitochondrial superoxide, of which Hoechst (Apexbio, Houston, USA) was applied to indicate nucleus of living cells. Cells in 96-well plates were washed twice with low glucose DMEM and the media were then replaced with DMEM containing 25 µM DCFH-DA. Cells were then incubated in the dark for 30 min, after which they were washed twice and resuspended in DMEM. The mouse eyes were enucleated after irradiation; retinas were then immediately frozen and homogenized in low glucose DMEM using a tissue grinder (KZ-II, Wuhan, China). The tissue homogenates were incubated with DCFH-DA (50 µM) at 37 °C in the dark for 30 min. Samples were then centrifuged at 3000 rpm for 5 min at 4 °C. Finally, the resulting pellets were washed twice with cold DMEM and then resuspended in low glucose DMEM. DCFH-DA fluorescence was measured at 488 nm of exciting light and 525 nm of emitting light using a FLUOstar OPTIMA Microplate Reader (BioTek Instruments, Inc., Winooski, VT, USA). As for MitoSOX Red staining, cells were incubated with 2 µM fluorochrome for 10 min, and then washed with PBS and examined using a fluorescence microscope (Leica, Wetzlar, Germany).

### Western blotting (WB)

R28 cells and retinas were harvested and the retinas were homogenized. The cells and homogenized retinas were then resuspended in ice-cold lysis buffer [1 M Tris-Cl (pH 6.8), 50% glycerol, and 10% SDS in ddH_2_O] containing a cocktail of protein phosphatase and protease inhibitors (Sigma–Aldrich). Whole samples were then subjected to sonication, clarified by centrifugation (12,000 rpm for 10 min), and the supernatant was collected. The supernatant was then mixed with 5× sample buffer and boiled at 98 °C for 10 min. The proteins in the lysates were separated by 12% sodium dodecyl sulfate-polyacrylamide gel electrophoresis (SDS-PAGE), and then transferred onto polyvinylidene fluoride (PVDF) membrane. After blocking with 5% bull serum albumin (BSA), the proteins on the membranes were immunoblotted overnight at 4 °C with the following primary antibodies: anti-Drp1 (1:1 000; #8570), anti-phospho Drp1 (Ser616) (1:1 000; #4494), anti-phospho Drp1 (Ser637) (1:1 000; #4867), anti-Cdk5 (1:1 000; #14145), and anti-MFF (1:1 000; #84580) antibodies, all of which were obtained from Cell Signaling Technology (Beverly, MA, USA). Anti-TTC11/FIS1 antibody was purchased from Abcam (1:200; ab71498, Cambridge, MA, USA). Anti-MID49 (1:800; 16413-1-AP) and anti-MID51 (1:1 000; 20164-1-AP) antibodies were purchased from proteintcch (Chicago, USA). Anti-β-actin (1:3 000; T0022) and anti-GAPDH (1:5 000; AF7021) antibodies were obtained from Affinity Biosciences (OH, USA). After three washes with PBST, the membranes were incubated with the appropriate horseradish peroxidase (HRP)-conjugated secondary antibody (1:10 000; Sigma–Aldrich) at room temperature for 1 h. Western blot bands were detected using an enhanced chemiluminescence solution (Millipore, Bedford, MA). Densitometric analysis was performed using Image J v1.8.0 software.

Six human peripheral blood samples were obtained, including two healthy controls, two negative controls (NC; received radiation treatment but did not develop RION), and two RION patients who received RT and were diagnosed at the Xiangya Hospital (Changsha, China). Importantly, NC and RION patients did not have any ocular surface disease, other optic nerve diseases, glaucoma, inflammatory disease, and evolutive ocular or other systemic diseases. Mononuclear cells were separated from blood samples from these patients; this process complied with our standard operation procedure. The resulting proteins were then subjected to Western blotting analysis. This clinical portion of the study was approved by the Ethics Committee of Xiangya Hospital of Central South University and written informed consent was obtained from all patients.

### Small interfering RNA (siRNA)

R28 cellular transfections were performed using Lipofectamine^®^ 2000 (Invitrogen Inc., Carlsbad, CA, USA) by following the manufacturer’s instructions. To silence Drp1 expression, siRNAs with the following sequences were used: 5′-GGUGCUAGGAUUUGUUAUATT-3′, 3′-UAUAACAAAUCCUAGCACCTT-5′ (synthesized by GenePharma, Shanghai, China). The sequence for Cdk5-RNAi was GGAGATCTGTCTACTCAAA (RiboBio, Guangzhou, China), and the negative control siRNA (ncRNA) was also constructed.

### Evaluation of mitochondria morphology

R28 cells from different experimental groups were fixed in 4% paraformaldehyde (Ncmbio, Suzhou, China) for 15 min. Cells were then washed three times with PBS and permeabilized with 0.1% Trixton-X-100 in PBS for 10 min. After blocking with 5% BSA for 30 min, cells were immunostained with an anti-Tom20 primary antibody (1:200; #42406, Cell Signaling Technology) with 5% BSA at 4 °C overnight. Following another three washes with PBS, cells were incubated with the appropriate secondary antibody of anti-rabbit IgG Alexa Fluor 488 (1:300; Sigma–Aldrich) with 5% BSA for 60 min. Cells were then washed five times with PBS and stained with 4′,6-diamidino-2-phenylindole (DAPI; Solarbio, Beijing, China). At least eight random images for each experimental condition were captured using a confocal laser scanning microscope (Leica). Transmission electron microscopy (TEM) was used to evaluate the mitochondria of RGCs in the mouse retinas using a HT7700 transmission electron microscope (Hitachi, Tokyo, Japan), by locating at RGC layer (Supplemental Fig. 5D). Briefly, BALB/c mice were anesthetized with chloral hydrate by i.p. injection and the eyes were quickly enucleated and fixed in 2.5% glutaraldehyde overnight at 4 °C. The processing of the samples and TEM imaging were performed following a standard protocol^23^. The average length of mitochondria in confocal images was analyzed by using Image J software. Approximately 70–150 mitochondria were measured in each image and the mean value was calculated, and five pictures from each group were randomly measured and analyzed; five high-magnification TEM images were randomly selected from each group, the area and perimeter of each mitochondrion in each image (about 8–15 mitochondria) were measured and the mean value were taken.

### Histopathologic examination

Paraffin-embedded retinal tissue sections (4 µm) were cut in vertical meridian through the optic disc, parallel to the maximum circumference of the eyeball. The sections were mounted onto microscope slides, deparaffinized, and stained with hematoxylin and eosin (HE). Micrographs of the stained retinas were generated using a light microscope (Leica). Retinal damage was evaluated by measuring the outer nuclear layer (ONL) thickness for irradiation dose selection. Low magnification images were taken for each group of the same multiple (Supplemental Fig. 5C), and we divided the position from the center of the optic nerve equidistantly with three straight lines perpendicular to the retina on both sides. Then the ONL thickness of each line was measured (six sets of data for each image were obtained) and averaged. The paraffin-embedded retinal tissue sections were then evaluated by immunofluorescence (IF) for Tuj1 (1:1 000; ab78078, Abcam). Results were obtained by Immunofluorescence slice scan (3D HISTECH, Budapest, Hungary).

### Detection of neuronal apoptosis

Apoptosis was assessed using a commercially available fluorometric terminal deoxynucleotidyl transferase dUTP nick end labeling (TUNEL) kit (Roche, Basel, Switzerland). Both R28 cells and paraffin sections of the mice eyes containing fragmented DNA were evaluated according to the kit manufacturer’s protocol. Apoptosis of R28 cells was also analyzed with flow cytometry on a FACS Canto II flow cytometer (BD Biosciences, NJ, USA). Annexin-V-FITC Apoptosis Detection Kit I (#556547, BD Pharmingen, USA) was used to assess apoptosis according to the manufacturer’s instructions.

### Statistical analysis

The data are presented as the mean ± standard deviation of results from at least three independent experiments. The statistical significance of the experimental differences between groups were analyzed using Student’s *t*-test, and comparisons among groups were analyzed using one-way analysis of variance (ANOVA) and Bonferroni’s multiple comparison test. All statistical analyses were performed on GraphPad Prism 8.0 (GraphPad Software, La Jolla, CA, USA). *P* values less than 0.05 were considered statistically significant and significance is indicated in the graphs by an asterisk. *P* values less than 0.01 and 0.001 are indicated by two and three asterisks, respectively.

## Results

### X-ray irradiation of R28 cells induced changes to mitochondrial dynamics and cell death

**Fig. 1:**
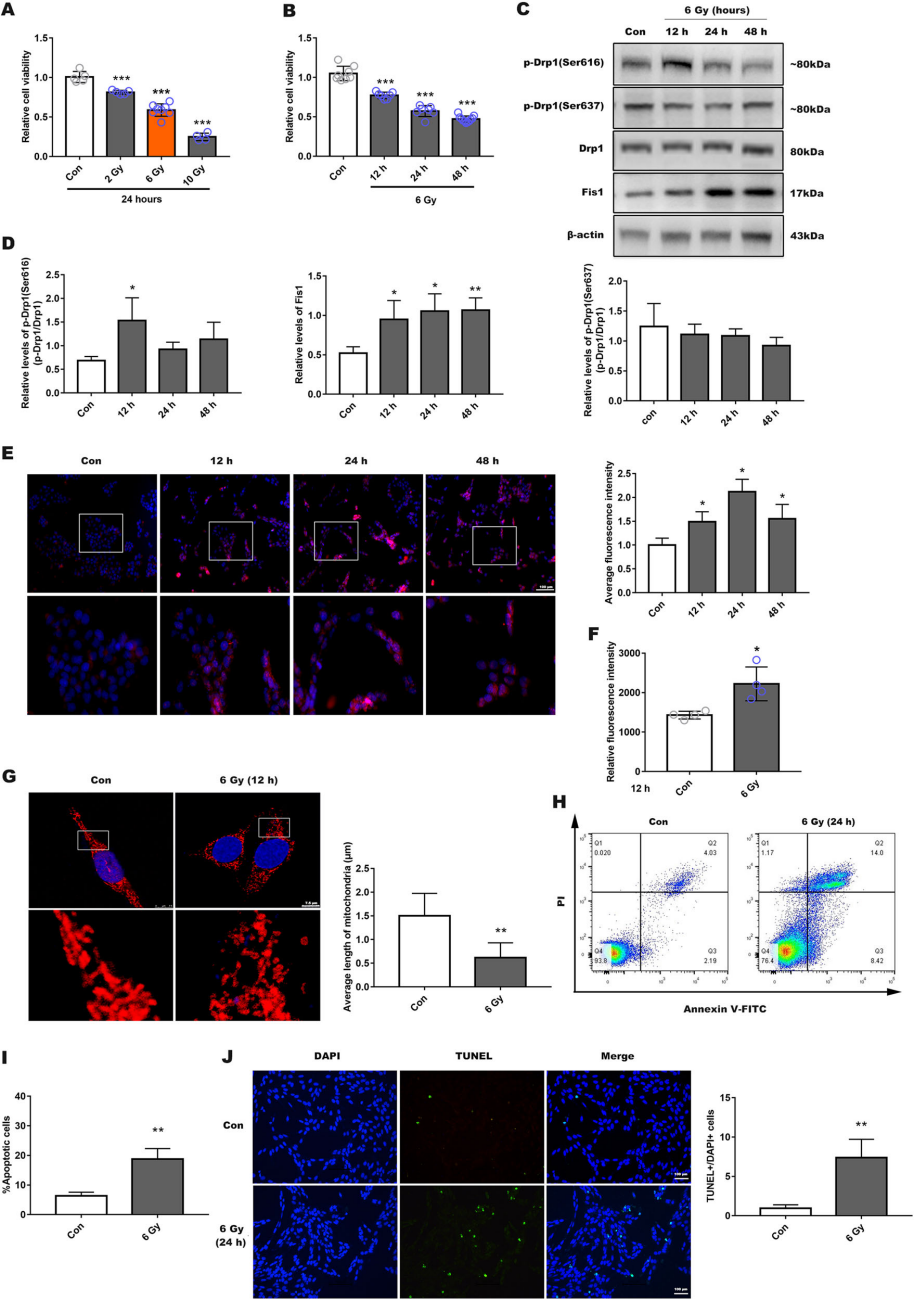
Radiation-induced mitochondrial dysfunction and apoptosis in R28 cells. **a** Cell viability was measured using a Cell Counting Kit-8 viability assay (CCK-8), in which R28 cells were exposed in different doses (2, 6, and 10 Gy) of X-ray for 24 h. **b** R28 cells were received 6 Gy X-ray for different times (12–48 h). **c** The levels of mitochondrial dynamics-related proteins were evaluated with 6 Gy radiation for 12, 24, or 48 h. **d** Quantitative analysis of protein expression. **e** Mitochondrial superoxide production was detected using MitoSOX Red at different point of time, and Hoechst (blue) indicated nucleus. Lower panel presents a fractionated gain of the image in the white rectangle of the upper panel. The average fluorescence intensity in each group was analyzed, scale bar is 100 µm. **f** Cellular ROS generation was analyzed by DCFH-DA using fluorescence intensity quantification. **g** Mitochondrial morphology identified using Tom20 antibodies by immunofluorescence after 12 h post-irradiation and observed by confocal microscopy. Lower panel is a fractionated gain of the upper image in the white rectangle, and average mitochondrial length was analyzed. Scale bar indicates 7.5 µm. **h** Apoptosis was detected using Annexin-V and PI staining via flow cytometry, 24 h post-irradiation, and the proportion of apoptotic cells was calculated (**i**). **j** R28 apoptosis also determined by TUNEL staining, bar is 100 µm. Data are presented as the mean ± SD (*N* = 3–7), *P < 0.05; **P < 0.01; ***P < 0.001.

We used CCK-8 assay to assess the appropriate dose of X-ray needed to induce death in ~50% of R28 cells. This threshold was reached after 24 h irradiation with 6 Gy, but 2 and 10 Gy irradiation achieved 20% and 75% cell death, respectively (Fig. 1a). There was also a time-dependent effect of radiation on cell viability (12, 24, and 48 h) (Fig. 1b and Supplemental Fig. 1A, B). As a result, 6 Gy of X-ray was used in subsequent cell experiments.

We next explored the specific changes in mitochondrial dynamics-related proteins after irradiation. WB was used to measure the expression levels of Drp1, Drp1 (Ser616), Drp1 (Ser637), and Fis1 at different time points (12–48 h). We found that Drp1 (Ser616) expression reached a peak at 12 h post-irradiation. The level of Fis1, associated with mitochondrial division, was increased. However, Drp1 and Drp1 (Ser637) expression remained unchanged (Fig. 1c, d). Simultaneously, we also observed the expression of other Drp1 receptors, including mitochondrial fission factor (MFF), mitochondrial dynamics of 49 kDa protein (MID49), and mitochondrial dynamics of 51 kDa protein (MID51)^24,25^. However, no significant expression changes were observed after irradiation (Supplemental Fig. 1C, D). Based on these findings, we performed subsequent experimental evaluations 12 h post-irradiation.

We evaluated intracellular ROS production and mitochondrial superoxide. Mitochondrial superoxide levels were increased at different time points post-irradiation (Fig. 1e), and cellular ROS production was also increased in the irradiated group (Fig. 1f). Mitochondrial outer membrane protein Tom20 was used to detect mitochondrial morphological changes. Immunofluorescence results showed that mitochondria became significantly more fragmented post-irradiation (Fig. 1g). In the control group, most of the mitochondria had a typical rod-shape and were elongated. Annexin-V/PI staining and TUNEL fluorescence both showed significant apoptosis at 24 h post-irradiation (Fig. 1h–j). These results confirmed that X-ray led to phosphorylation of Drp1 (Ser616), mitochondrial fragmentation and free radical damage in R28 cells, causing cells death ultimately.

### Mdivi-1 and Drp1 siRNA treatment ameliorated radiation-induced damage to R28 cells

**Fig. 2:**
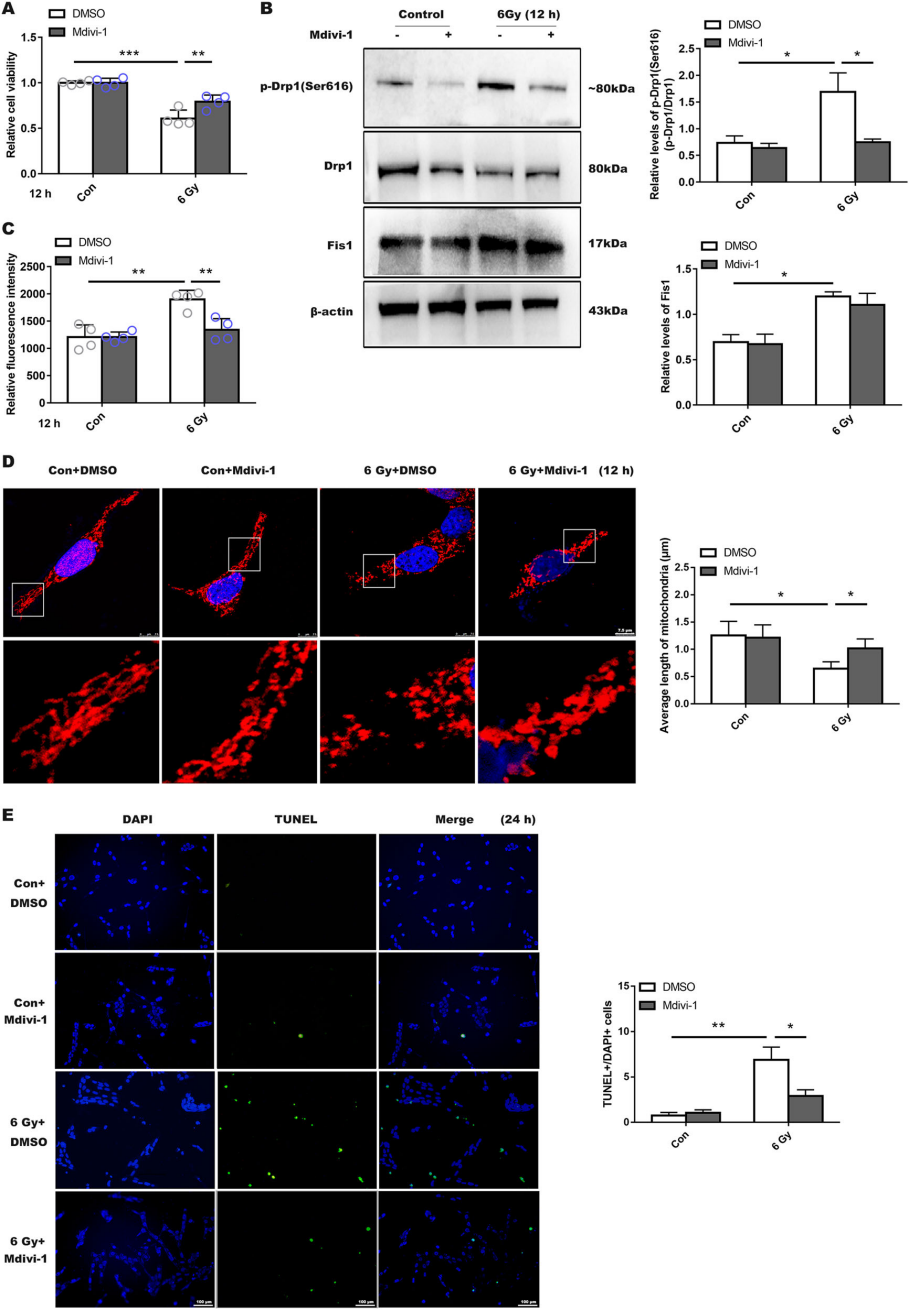
Inhibition of Drp1 activity affected radiation-induced mitochondrial fragmentation and R28 cells death. **a** Higher Cell viability with Mdivi-1 treated in advance after 12 h of 6 Gy irradiation. **b** Drp1 (Ser616), Drp1 (Ser637), Drp1, and Fis1 expression levels were evaluated in R28 incubated with Mdivi-1 (10 µmol). **c** ROS production was detected after Mdivi-1 treatment. **d** Mitochondrial morphology changes as shown by immunofluorescence. **e** Cells apoptosis was observed by TUNEL staining after irradiation for 24 h. *Y-*axis shows the ratio of TUNEL staining positive numbers/total DAPI staining cells numbers after treatment with Mdivi-1. Data are presented as the mean ± SD (*n* = 3–7), *P < 0.05; **P < 0.01; ***P < 0.001.

Based on the effective concentration reported in the literature for the putative Drp1 inhibitor mdivi-1^26,27^, 5 and 10 µmol of mdivi-1 were used. siRNA was designed to effectively silence Drp1 expression, and the silencing effect of siRNA was verified before the sequence 1 was used (Supplemental Fig. 2B). We found that the irradiated group treated with a low concentration of mdivi-1 (5 µmol) had a significant decrease in cell viability compared to control group (Supplemental Fig. 2A). However, the group pretreated with 10 µmol mdivi-1 showed a notably protective effect (Fig. 2a). Thus, we used 10 µmol as an effective concentration in the following experiments. WB results showed Drp1 (Ser616) expression in the irradiated group treated with mdivi-1 was significantly lower than that in untreated group (Fig. 2b). Furthermore, ROS production in the treated group was significantly lower than that in untreated group (Fig. 2c). Immunofluorescence results showed that the mitochondrial fragmentation in the mdivi-1 treated group was not as severe as that observed in the untreated irradiation group (Fig. 2d).

**Fig. 3:**
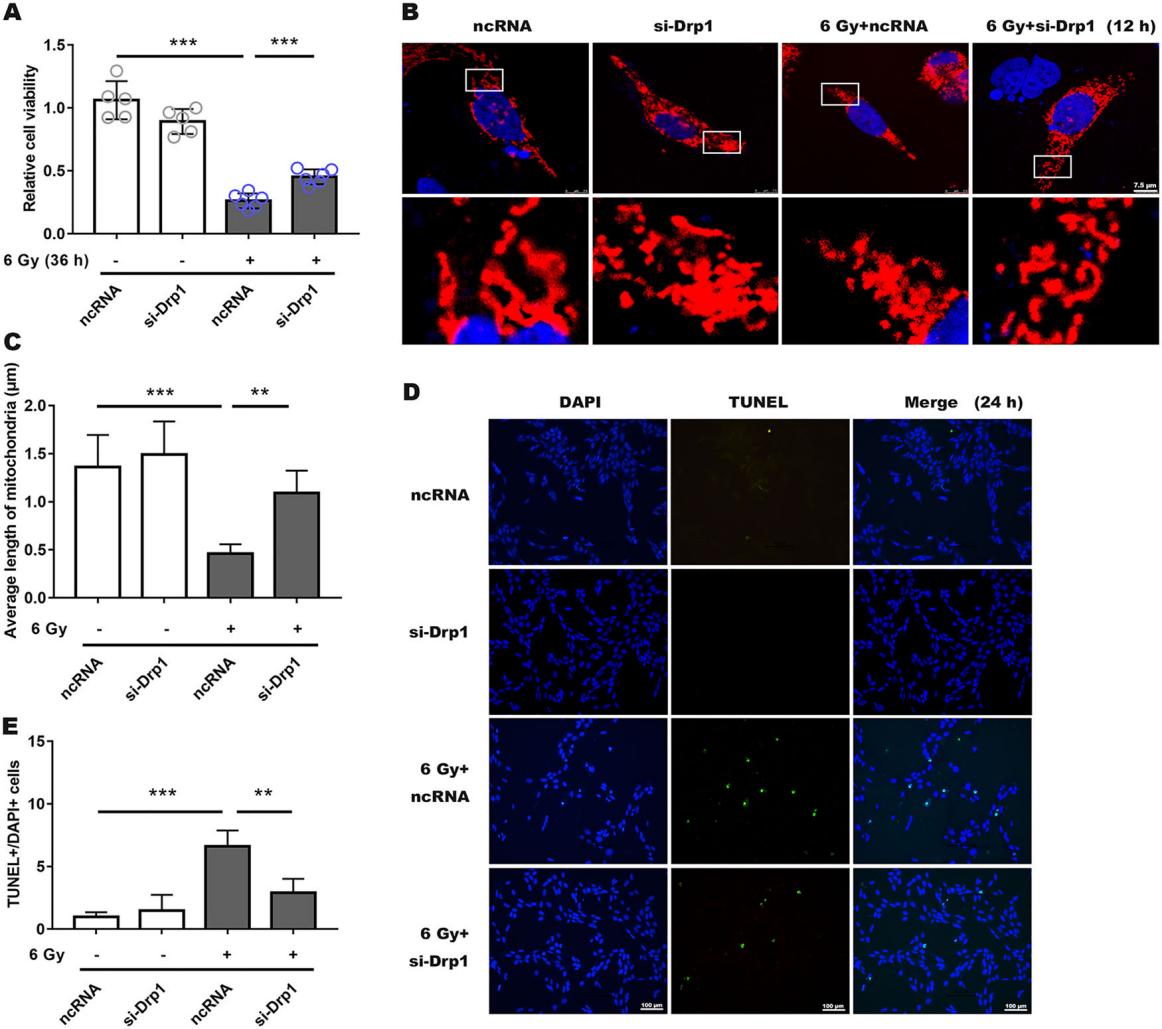
Silencing Drp1 expression by siRNA ameliorated radiation-induced damage to R28 cells. **a** Cell viability was measured following a 36 h of 6 Gy radiation, which was treated with siRNA and ncRNA. **b** Mitochondrial morphology had a notably prolonged shape after prior siRNA incubation in irradiation group. **c** Average mitochondrial length was measured in each group. **d, e** Decreased apoptotic RGCs in the siRNA-treated group after irradiation were measured using TUNEL fluorescence. Data are presented as the mean ± SD (*n* = 3–6), *P < 0.05; **P < 0.01; ***P < 0.001.

Moreover, when compared with the ncRNA transfected irradiated group, si-Drp1 revealed a protective effect in terms of cell viability (Fig. 3a), and mitochondrial morphology was significantly lengthened and the average mitochondrial length was significantly increased (Fig. 3b, c), while ROS generation was not significantly different from that in the ncRNA-irradiated group, and both were significantly elevated (Supplemental Fig. 2C). Similarly, for groups treated with mdivi-1 or transfected with siRNA, the number of apoptotic cells were significantly less (Figs. 2e and 3d, e; Supplemental Fig. 3B and Supplemental Fig. 4B, C), indicating that both mdivi-1 and si-Drp1 promoted R28 cells survival following X-ray exposure.

### Cdk5-mediated phosphorylation of Drp1 at serine 616 regulated mitochondrial dynamics during radiation-induced injury in R28 cells

**Fig. 4:**
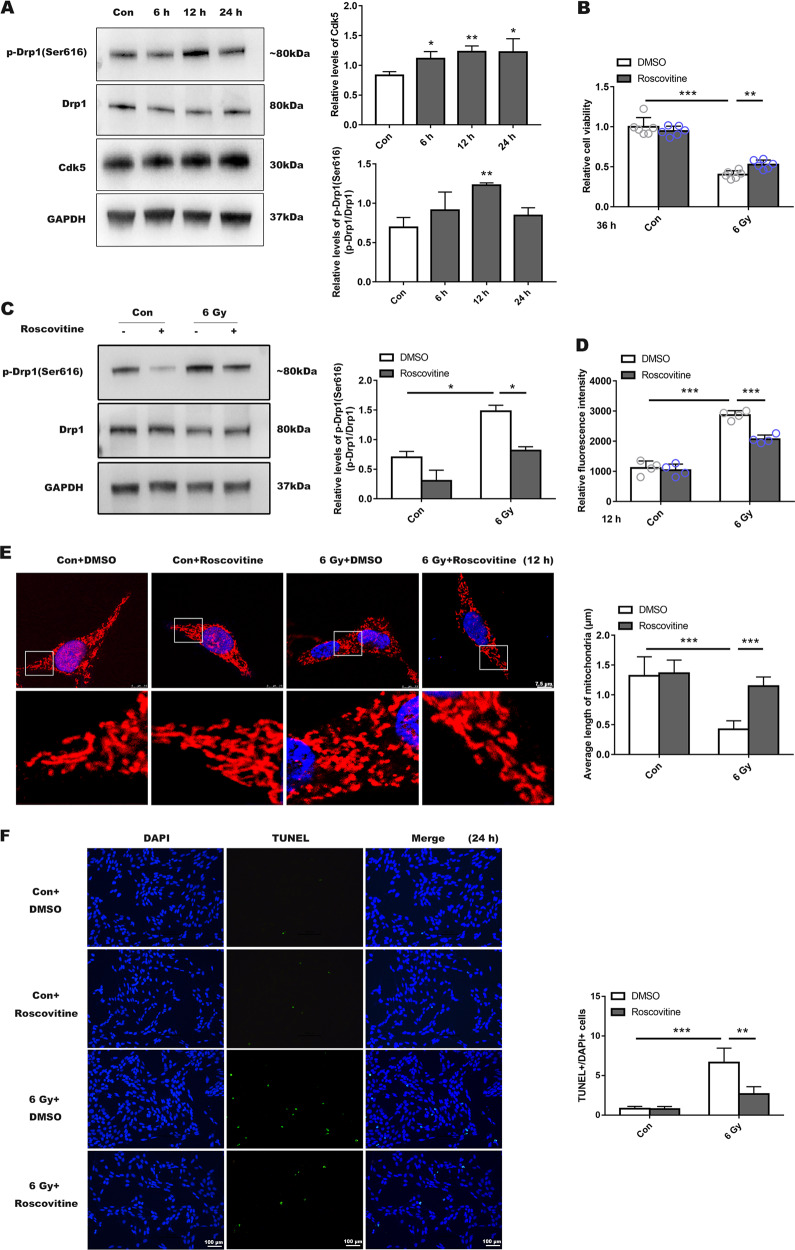
Drp1 is directly phosphorylated by Cdk5 at serine 616 and inhibition of Cdk5 activation is beneficial to mitochondrial morphology and cellular survival. **a** According to WB results, Drp1 is a direct substrate of Cdk5. All protein bands were normalized to loading control, GAPDH. Bar graphs represent relative levels of these proteins. **b** Cell viability measured after roscovitine intervention (inhibitor of Cdk5). **c** Levels of Drp1 (Ser616), Drp1 were detected after 6 Gy radiation with pre-treatment using roscovitine. **d** Roscovitine reduced ROS generation after irradiation. **e** Confocal images of mitochondrial morphology in the different groups. **f** TUNEL results after roscovitine treatment. Data are presented as the mean ± SD (*n* = 3–6), *P < 0.05; **P < 0.01; ***P < 0.001.

**Fig. 5:**
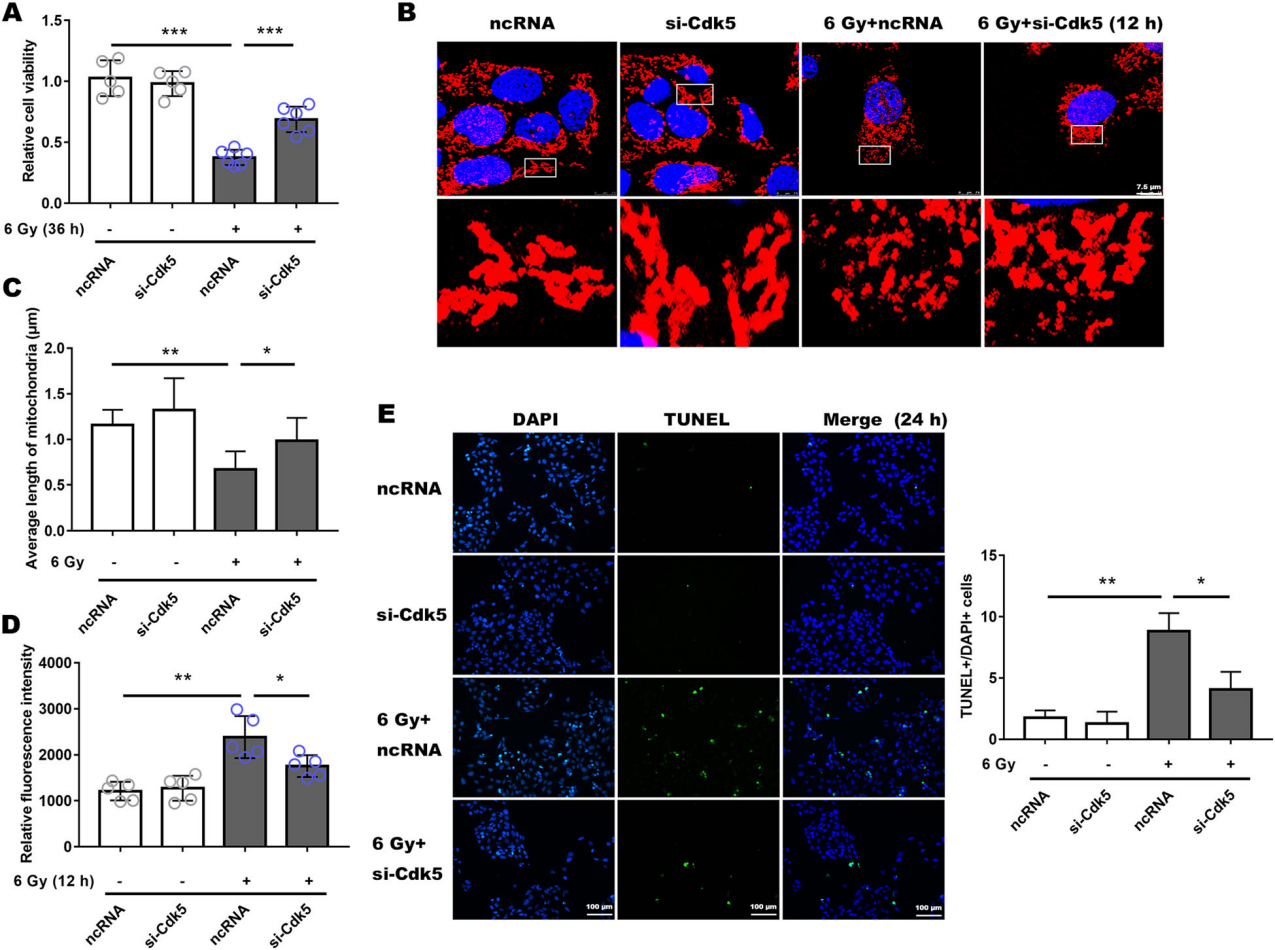
Silencing Cdk5 expression could reduce radiation-induced injury to R28. **a** Cell viability was detected after 36 h of 6 Gy radiation and mitochondrial morphology (**b,** **c**), also ROS level (**d**) and apoptotic cells in each group (**e**) were presented. Data are presented as the mean ± SD (*n* = 3–6), *P < 0.05; **P < 0.01; ***P < 0.001.

We evaluated the expression of Cdk5 by extracting total cellular protein at 6, 12, and 24 h post-irradiation. Cdk5 expression increased over time when compared with control (Fig. 4a). Next, we used roscovitine (the classic Cdk5 inhibitor) at 10, 15, and 20 µmol to treat R28. Our results demonstrated that only 15 µmol roscovitine provided a distinct protective effect, with higher concentrations being toxic to the cells (Fig. 4b and Supplemental Fig. 3A). After treatment with roscovitine (15 µmol), previously observed upregulation of Drp1 (Ser616) was suppressed (Fig. 4c), ROS production significantly decreased (Fig. 4d) and the cells treated with roscovitine showed a significant elongation of mitochondria (Fig. 4e), and the number of apoptotic cells was reduced as well (Fig. 4f and Supplemental Fig. 3B), indicating that roscovitine could increase the survival rate of R28 cells. We also used si-Cdk5 to investigate the specific role of Cdk5 in radiation injury. We chose sequence 1 for the following experiments based on WB results (Supplemental Fig. 4A). Silencing Cdk5 showed the same effect in mitigating impaired phenotype as Roscovitine (Fig. 5a–e and Supplemental Fig. 4B, C).

### In vivo X-rays induced retinal morphology changes and damage to visual functioning

**Fig. 6:**
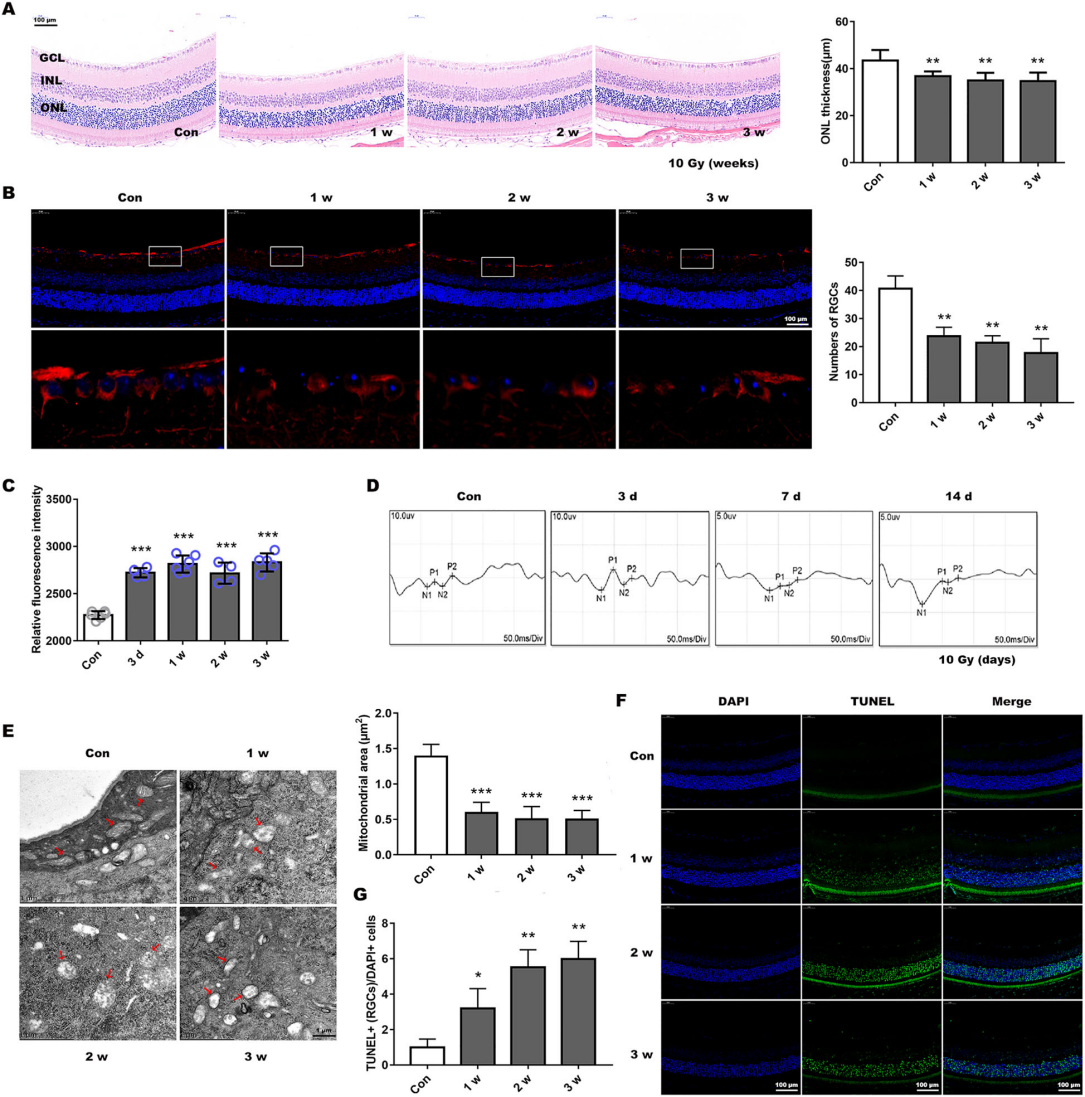
X-ray-induced mitochondrial dynamic changes and visual function damage in BALB/c mice. **a** Hematoxylin and eosin staining showed retinal structures changes after 10 Gy exposure and analyzed the outer nuclear layer (ONL) thickness. GCL ganglion cell layer, INL inner nuclear layer. Scale bar is 100 µm. **b** RGCs and neuronal axons identified using Tuj1 antibodies by immunofluorescence with retinal paraffin sections. Lower panel presents a fractionated gain of the image in the white rectangle of the upper panel. The number of RGCs at different post-radiation time (1, 2, and 3 weeks) were then calculated. Bar, 100 µm. **c** Retinal ROS generation was determined by DCFH-DA fluorescence intensity quantification after radiation for 3 d or 1–3 weeks. **d** Visual function was evaluated by VEP recording after 3, 7, or 14 d irradiation. **e** Mitochondrial micromorphology was determined by transmission electron microscopy. The red arrows indicate markedly morphologic mitochondria, and mitochondrial area was measured. Bar, 1 µm. **f** Retinal paraffin sections were stained by TUNEL to observe cellular apoptosis, and **g** TUNEL-positive RGCs were counted. Bar is 100 µm. Data are presented as the mean ± SD (*n* = 3–6), *P < 0.05; **P < 0.01; ***P < 0.001.

BALB/c mice were irradiated with X-ray (0, 8, 10, or 12 Gy) to induce RION within a short time frame^28^. At 1, 2, or 3 weeks post-irradiation, the eyes were processed for HE staining to determine the appropriate radiation dose. Our results indicated that ONL thickness was significantly thinner at different time points after both 10 and 12 Gy irradiation, while 8 Gy did not cause obvious changes at 1 week post-irradiation (Fig. 6a and Supplemental Fig. 5A, B). Therefore, we chose a smaller effective dose of 10 Gy for all subsequent experiments.

Beta-III-tubulin (Tuj1), expressed in cytoplasm and axons of active RGCs, is a typical marker used to detect RGCs^29^. Tuj1 immunostaining revealed a reduction in the number of ganglion cells and neuronal axons in the mouse retinas after irradiation (Fig. 6b). And retinal ROS production was evaluated at 3 d as well as at 1, 2, and 3 weeks, these levels were all significantly increased (Fig. 6c). VEP latency reflects the velocity of signal conduction along the visual pathway and is considered to be an accurate measurement of pathological changes in the optic nerve^30^. Comparatively, VEP amplitude is considered to be closely correlated with axonal damage of RGCs^31^. Therefore, the mice were subjected to VEP testing at 3, 7, and 14 d (visual function changes before structural damage). Results showed there were significant delays in P1 and P2 waves at different time points post-irradiation, moreover, amplitude also decreased (Fig. 6d and Table 1), implying that visual function was impaired.

**Table 1:** Comparison of P1-N2-P2 parameters of VEP before and after X-ray radiation.

	Day 0	Day 3	Day 7	Day 14
*10 Gy*				
Latency, ms				
P1	72.6 ± 5.3	81.0 ± 5.7	94.3 ± 4.6*	100.3 ± ± 3.8**
P2	98.7 ± 3.3	114.2 ± 3.5**	123.8 ± 4.7**	128.8 ± 4.6**
Amplitude, uv				
N2-P2	4.1 ± 0.4	1.5 ± 0.2**	0.8 ± 0.1***	1.0 ± 0.2***

Data are presented as the mean ± SD (*N* = 5).

*P < 0.05.

**P < 0.01.

***P < 0.001.

Using TEM to evaluate mitochondrial changes in RGCs, we found that the mitochondria in the irradiated group had obvious structural changes, appeared swollen with disordered or absent cristae structure, besides mitochondrial area and perimeter also showed reduction (Fig. 6e and Supplemental Fig. 5E). TUNEL staining indicated that the number of apoptotic cells in the retinal ganglion cell layer increased significantly after irradiation (Fig. 6f, g).

### Mitochondrial dynamics changed after irradiation and the potential in vivo therapeutic target of RION

**Fig. 7:**
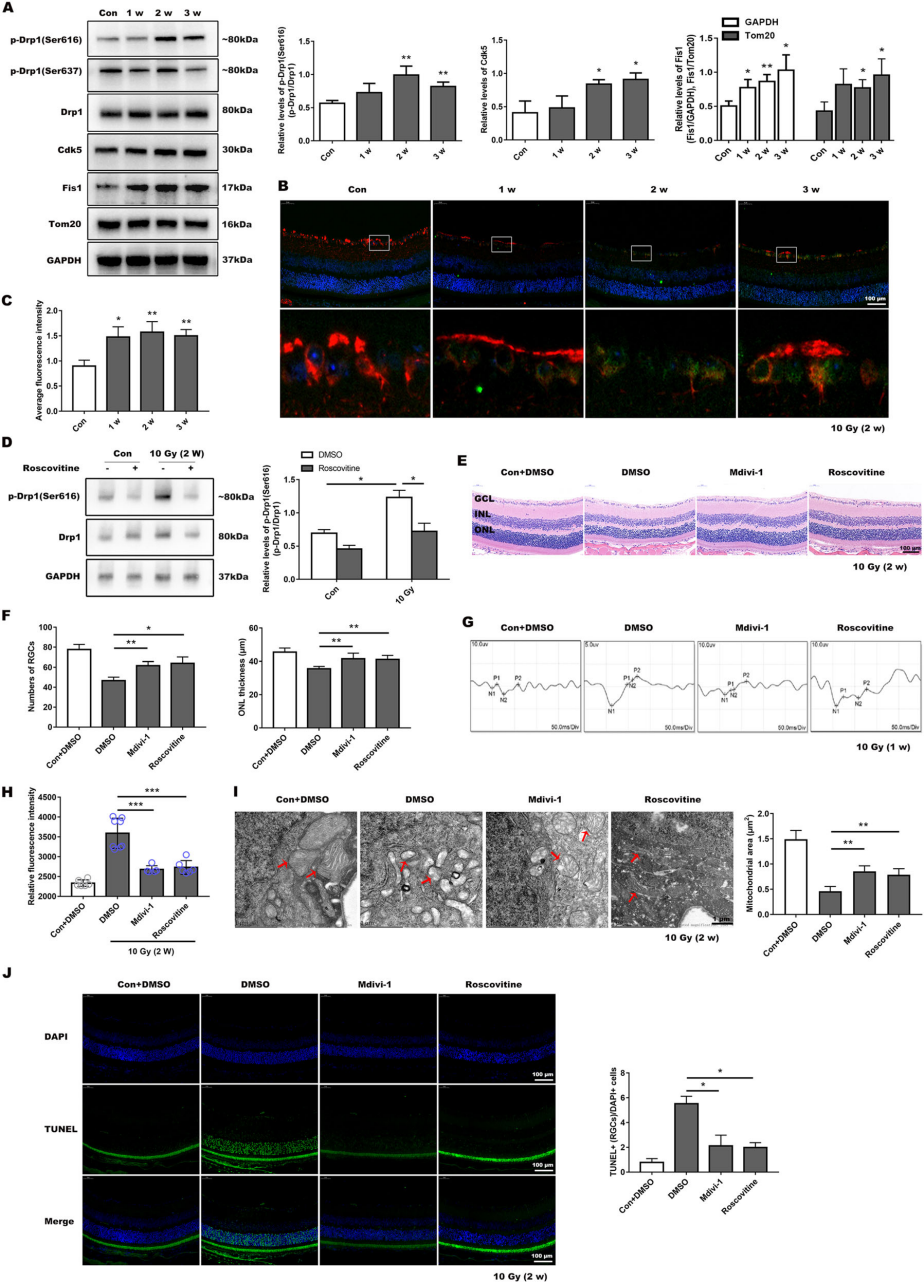
Both Mdivi-1 and roscovitine protected visual function and reduced cell death in irradiated mice. **a** Expression levels of mitochondrial dynamics-related proteins after irradiation for 1–3 weeks and the mitochondrial structural protein Tom20 was also used as a loading control. Quantitative protein statistics was conducted. **b** Tuj1 (red) and Drp1 (Ser616) (green) were co-stained by immunofluorescence, and **c** average fluorescence intensity of Drp1 (Ser616) in RGCs was analyzed using image J. Bar, 100 µm. **d** Upregulation of p-Drp1 (Ser616)/Drp1 after radiation for 2 weeks was suppressed by roscovitine injection. **e** HE staining showed retinal structures in different groups after two weeks post-irradiation, and **f** numbers of RGCs, ONL thickness were measured. **g** VEP recordings of Mdivi-1- or roscovitine-treated groups after 1 week of radiation. **h** ROS production was detected after X-ray exposure for 2 weeks with Mdivi-1 or roscovitine treatment. **i** Mitochondrial morphology was determined using TEM following Mdivi-1 and Roscovitine intervention. **j** Following treatment with two inhibitors, TUNEL-positive RGCs reduced in the irradiated group. Data are presented as the mean ± SD (*n* = 3–6), *P < 0.05; **P < 0.01; ***P < 0.001.

We detected the expression of mitochondrial dynamics-related proteins in the retina of mice at 1, 2, and 3 weeks post-irradiation. We found that Drp1 (Ser616) expression increased at the second and third week. Comparatively, Drp1 (Ser637) expression was essentially unchanged. Fis1 and Cdk5 expression in the irradiated group was significantly higher (Fig. 7a). Meanwhile we co-stained Tuj1 and Drp1 (Ser616) for immunofluorescence analysis, and results showed that the fluorescence intensity of Drp1 (Ser616) was significantly increased post-radiation in RGCs (Fig. 7b, c). After the roscovitine injection, the retinal level of Drp1 (Ser616)/Drp1 was suppressed at 2 weeks post-radiation (Fig. 7d), suggesting that Cdk5 may be the upstream regulator of Drp1-mediated mitochondrial dysfunction.

We also used mdivi-1 and roscovitine in mice to investigate if inhibition of the Cdk5-mediated Drp1 (Ser616) pathway would ameliorate retinal morphology damage and subsequent injury to visual functioning. There were apparent decreased RGCs numbers and ONL thinning in the untreated irradiated group at 2 weeks post-irradiation, while no distinct decrease in either the mdivi-1-treated or roscovitine-treated group according to HE results (Fig. 7e, f). We then recorded the VEP results 1 week after irradiation, and found that delay of P1 and P2 waves was evident in the untreated irradiated group compared with inhibitors treated group (Fig. 7g and Table 2). And there were distinct ROS production decreases in these treated groups (Fig. 7h). TEM indicated that Mdivi-1 and Roscovitine protected mitochondrial structural morphology following irradiation, since the number of mitochondria with disorganized structure was lower in these groups, and the mitochondrial area and perimeter were added (Fig. 7i and Supplemental Fig. 5F). The numbers of TUNEL-positive RGCs and other retinal cells were much lower following injection of inhibitors (Fig. 7j). These findings demonstrated that inhibition of the Cdk5-mediated Drp1 (Ser616) pathway could alleviate the radiation-induced damage to mouse retina and improve visual function.

**Table 2:** Comparison of P1-N2-P2 parameters of VEP 7 days after X-ray radiation across different groups.

	Control	10 Gy+DMSO	10 Gy+Mdivi-1	10 Gy+Roscovitine
*10 Gy*				
Latency, ms				
P1	54.3 ± 1.3	90.5 ± 4.8	69.9 ± 6.8*	80.1 ± 5.1
P2	109.6 ± 2.8	130.5 ± 2.4	106.6 ± 6.0*	125.0 ± 2.5
Amplitude, uv				
N2-P2	5.8 ± 0.9	1.4 ± 0.2	5.1 ± 1.3*	4.8 ± 1.1*

Data are presented as the mean ± SD (*N* = 5).

*P < 0.05.

**P < 0.01.

***P < 0.001.

Moreover, we collected six human peripheral venous blood samples (two controls, two negative controls (NC), and two RION patients), and mononuclear cells were extracted and subjected to electrophoresis and WB to assess the various proteins involved in the Cdk5/Drp1 signaling pathway. The results showed upregulation of Drp1 (Ser616) and Fis1 in RION patients’ peripheral venous blood (Supplemental Fig. 6), consistent with our in vivo and in vitro results. Collectively, our findings provide corroborating evidence for the role of the Cdk5/Drp1 signaling pathway in RION development.

## Discussion

The goal of RT is to deliver as much of the maximum therapeutic dose as possible to the lesion, while minimizing radiation to the adjacent tissue. However, the effective therapeutic dose required for RT is generally higher than that for normal tissue, leading to delayed tissue necrosis^32^. Ionizing radiation often damages the optic nerve by primarily generating injuries to the neurons and their axons, and mainly damages RGCs, which eventually results in irreversible loss of vision. In our study, we found that after exposed to X-ray, the irradiated cells exhibited obvious morphological changes and growth inhibition. The severity of visual impairment can range from subclinical changes in visual-evoked potentials to complete blindness^33^. Accordingly, we found that irradiated mice showed VEP waves delay and decreased amplitude, suggesting that the visual function of these mice was significantly impaired.

Mitochondrial dynamics plays an important role in cell growth, redistribution of mitochondria, and maintenance of normal mitochondrial networks^34^. Critical molecules in these processes were initially identified by genetic analysis of flies and yeast and belong to the guanosine triphosphatase (GTPase) dynamin-related proteins (DRPs)^35^. More specifically, these proteins are involved in mitochondrial division and fusion. Among them, Drp1 is mainly involved in mitochondrial division. Drp1 is distributed in the cytoplasm, and when cells are exposed to external stimulus, Drp1 would recruit to the outer mitochondrial membrane and binds with Fis1. The mitochondria are then split into two parts by hydrolysis of GTPase^36^. We analyzed protein samples from cells and mouse retinas, and found Drp1 (Ser616)/Drp1 and Fis1 levels remarkably increased after irradiation. Furthermore, we found mitochondrial morphology was significantly changed and the number of apoptotic cells increased after irradiation. These results suggest that either in vitro or in vivo radiation-mediated mitochondrial kinetic abnormalities occurred primarily by Drp1 (Ser616) phosphorylation, which interacted with Fis1 to induce mitochondrial dysfunction and/or disrupted membrane integrity. When mitochondria are severely damaged, fission becomes the dominant process to remove damaged mitochondria. The fission GTPase Drp1 promotes Bax-dependent cytochrome c redistribution from mitochondria to the cytoplasm—an event that initiates the activation of caspase protease executioners^37^. Past studies have demonstrated that inhibiting Drp1 expression or its function can ameliorate mitochondrial division, and play a protective role in multiple diseases ranging from heart disease to neurodegenerative disorders^38,39^. Mdivi-1 is a small-molecule inhibitor of mitochondrial fission that specifically targets Drp1^40,41^. Studies have reported that mdivi-1 can reversibly inhibit mitochondrial complex I^42^, and reduce generation of oxygen free radicals in cells and tissues^26,27^. In our study, Mdivi-1 was shown to inhibit Drp1 phosphorylation and also decrease ROS production, thereby rescuing mitochondrial fission phenotype, increasing cellular survival and improving the visual function of mice. However, no significant difference was observed in mitochondrial morphology between DMSO and Mdivi-1 control groups. It has been shown that mitochondrial fusion events primarily produce elongated mitochondria^43^, while in the current study, Mdivi-1 did not impact the mitochondrial fusion process. Under normal conditions, Drp1 in R28 cells or ganglion cells has a low function in regulating mitochondrial division, and only increasing Drp1 activity can induce division, while Mdivi-1 can decrease mitochondrial fission by inhibiting Drp1. Hence, it is reasonable that there was no obvious mitochondrial elongation in the intervention control group. Also, we found ROS levels were not significantly decreased in the si-Drp1 irradiated group. Given this, we speculated that ROS was a direct production of ionizing radiation, and ROS generated by Drp1 activity is not predominant. While the effects of Mdivi-1 alleviating mitochondrial fission and cell apoptosis were apparent.

In previous studies, Drp1 (Ser616) is phosphorylated by Cdk1 in a specific environment, but Cdk1 is expressed at lower levels in nervous tissue^44^. In addition, Cdk5 is primarily expressed in neurons, is capable of phosphorylating Drp1 (Ser616), and is involved in the regulation of mitochondrial dynamics^45^. Cdk5 is mainly distributed in mitotic neurons and participates in biological processes such as neuronal cytoskeletal reconstruction, oxidative stress, and apoptosis^46^. WB results indicated that Cdk5 expression levels were significantly increased after irradiation, and after inhibitor (roscovitine) of Cdk5 was used, it was observed that Drp1 (Ser616) upregulation after radiation was suppressed. Importantly, the level of ROS production, excessive mitochondrial fragmentation and the number of apoptotic cells decreased both in vivo and in vitro. Also, the visual function of the mice after treatment improved dramatically, and Cdk5-RNAi exerted similar protective effect. Based on these results, we believe that Cdk5 is an important regulator of abnormal, Drp1-induced mitochondrial dynamics and roscovitine can protect cells and tissues from radiation-induced damage. Previous studies have reported that the upstream molecules of different stimuli or models that mediate Drp1 activity include Erk, calcineurin, and protein kinase A (PKA)^47–49^. However, our study demonstrated that Cdk5 and Drp1 (Ser616) levels were notably increased after irradiation. The fact that a Ckd5 inhibitor was used to intervene as a protective approach provides support for targeting Cdk5. We also have mentioned above that radiation can cause DNA damage; studies have reported that in neurons, Cdk5 is hyperactivated upon oxidative stress and DNA damage^50^, therefore these factors may be the essential part in activation of Cdk5 under radiation exposure.

**Fig. 8:**
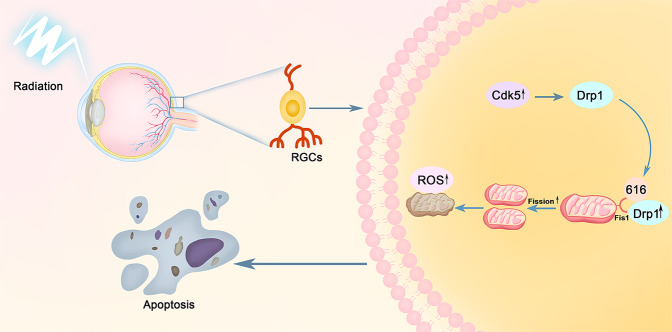
The putative signaling pathways of X-ray induced RGCs apoptosis by altering its mitochondrial dynamic balance. Radiation-induced optic neuropathy (RION) involves damage to the optic nerve, and mainly resulting in retinal ganglion cells (RGCs) injury. Cdk5 kinase activation is the trigger of the apoptotic process, resulting in Drp1 phosphorylation at serine 616. Increased p-Drp1 and then recruited and translocated from the cytoplasm to the outer mitochondrial membrane where it combines Fis1 and leads to mitochondrial division. A large number of ROS are produced simultaneously and results in RGCs apoptosis ultimately.

Numerous studies have shown that ionizing radiation causes a massive production of neuronal ROS, which provokes oxidative stress and leads to neuronal damage. Shirai and Parihar et al. found that exposure to X-rays (2–8 Gy) resulted in a reduction in the length, number, and regional distribution of dendrites in hippocampal neurons. The number of dendritic spines and their density were also significantly decreased, with these changes associated with the oxidative stress response to ionizing radiation^51,52^. Our results showed that ROS production in irradiated R28 cells and the retinas of irradiated mice was significantly higher than controls. ROS levels increased at various time points after irradiation and began earlier than increased Drp1 (Ser616). This indicated that ROS were involved in a series of damage events and mitochondrial abnormalities following the occurrence and development periods of irradiation, and may be one of the upstream regulator of Cdk5/Drp1 pathway. We presented the specific schematic diagram of our research in Fig. 8. Yamamori et al. showed that irradiation caused mitochondrial ROS production, which was attributed to upregulated mitochondrial ETC function and mitochondrial content. Moreover, irradiation-induced cell-cycle arrest contributed to an increase in the level of mitochondrial ROS by accumulating cells in the G2/M phase^53^. This provides a possible explanation for the generation of ROS after irradiation.

In conclusion, we have provided evidence for a specific signal pathway involved in retinal neuronal cell damage and visual function impairment in mice following X-ray irradiation. Radiation may cause increased ROS production inside and outside the cell, simultaneously activating Cdk5 and then regulating the phosphorylation of Drp1 at serine 616. Drp1 phosphorylation may therefore mediate mitochondrial fission, initiate programmed cell death, and result in irreversible damage to cells and tissues. Our study provides a clear direction and theoretical basis for better elucidating RION pathogenesis. Further research is needed to more specifically explore potential mechanisms such as the role of mitochondrial autophagy and mitochondrial complex function in RION, to improve our understanding of the mitochondrial component in radiation-induced damages, and to provide new ideas for the clinical diagnosis and treatment of RION.

## Supplementary information

Supplementary figure legends

Supplemental Figure 1

Supplemental Figure 2

Supplemental Figure 3

Supplemental Figure 4

Supplemental Figure 5

Supplemental Figure 6

## References

[1] Jeganathan VSE, Wirth A, MacManus MP (2011). Ocular risks from orbital and periorbital radiation therapy: a critical review. Int. J. Radiat. Oncol..

[2] Pollock BE, Link MJ, Leavitt JA, Stafford SL (2014). Dose-volume analysis of radiation-induced optic neuropathy after single-fraction stereotactic radiosurgery. Neurosurgery.

[3] Indaram M, Ali FS, Levin MH (2015). In search of a treatment for radiation-induced optic neuropathy. Curr. Treat. Options Neurol.

[4] Parihar VK (2015). Persistent changes in neuronal structure and synaptic plasticity caused by proton irradiation. Brain Struct. Funct..

[5] Gaddini L (2018). Exposing primary rat retina cell cultures to γ-rays: an in vitro model for evaluating radiation responses. Exp. Eye Res..

[6] Bo T (2018). Calmodulin-dependent protein kinase II (CaMKII) mediates radiation-induced mitochondrial fission by regulating the phosphorylation of dynamin-related protein 1 (Drp1) at serine 616. Biochem. Biophys. Res. Commun..

[7] Kempf SJ (2015). Low-dose ionizing radiation rapidly affects mitochondrial and synaptic signaling pathways in murine hippocampus and cortex. J. Proteome Res..

[8] Tohru Yamamori SITB (2015). Inhibition of the mitochondrial fission protein dynamin-related protein 1 (Drp1) impairs mitochondrial fission and mitotic catastrophe after x-irradiation. Mol. Biol. Cell.

[9] Huang CY (2018). HMGB1 promotes ERK-mediated mitochondrial Drp1 phosphorylation for chemoresistance through RAGE in colorectal cancer. Cell Death Dis..

[10] Zaja I (2014). Cdk1, PKCδ and calcineurin-mediated Drp1 pathway contributes to mitochondrial fission-induced cardiomyocyte death. Biochem Biophys. Res.Commun..

[11] Zhang C, Huang J, An W (2017). Hepatic stimulator substance resists hepatic ischemia/reperfusion injury by regulating Drp1 translocation and activation. Hepatology.

[12] Chang C, Blackstone C (2007). Cyclic AMP-dependent protein kinase phosphorylation of Drp1 regulates Its GTPase activity and mitochondrial morphology. J. Biol. Chem..

[13] Kam WW, Banati RB (2013). Effects of ionizing radiation on mitochondria. Free Radic. Biol. Med..

[14] Lafargue A (2017). Ionizing radiation induces long-term senescence in endothelial cells through mitochondrial respiratory complex II dysfunction and superoxide generation. Free Radic. Biol. Med..

[15] Barber AJ (2001). Insulin rescues retinal neurons from apoptosis by a phosphatidylinositol 3-kinase/Akt-mediated mechanism that reduces the activation of caspase-3. J. Biol. Chem..

[16] Núñez-Álvarez C, Osborne NN (2019). Blue light exacerbates and red light counteracts negative insults to retinal ganglion cells in situ and R28 cells in vitro. Neurochem. Int..

[17] Fox TE (2006). Diabetes alters sphingolipid metabolism in the retina: a potential mechanism of cell death in diabetic retinopathy. Diabetes.

[18] Deng S (2018). Mitochondrial dynamics and protective effects of a mitochondrial division inhibitor, Mdivi-1, in lipopolysaccharide-induced brain damage. Biochem. Biophys. Res. Commun..

[19] Wu Q (2016). Mitochondrial division inhibitor 1 (Mdivi-1) offers neuroprotection through diminishing cell death and improving functional outcome in a mouse model of traumatic brain injury. Brain Res..

[20] Li L, Zhang C, Zi X, Tu Q, Guo K (2015). Epigenetic modulation of Cdk5 contributes to memory deficiency induced by amyloid fibrils. Exp. Brain Res..

[21] SALLAM H (2008). Age-dependent pharmacokinetics and effect of roscovitine on Cdk5 and Erk1/2 in the rat brain. Pharmacol. Res..

[22] Zhang Z, Liu L, Wu S, Xing D (2016). Drp1, Mff, Fis1, and MiD51 are coordinated to mediate mitochondrial fission during UV irradiation‐induced apoptosis.. FASEB J..

[23] Raghav K (2018). Structural basis of mitochondrial receptor binding and constriction by DRP1. Nature.

[24] Jiang B (2013). Intravitreal transplantation of human umbilical cord blood stem cells protects rats from traumatic optic neuropathy. PLoS ONE.

[25] Deng G (2018). BMP4 promotes hepatocellular carcinoma proliferation by autophagy activation through JNK1-mediated Bcl-2 phosphorylation. J. Exp. Clin. Cancer Res..

[26] Rosdah AA (2017). Mdivi-1 protects human W8B2+ cardiac stem cells from oxidative stress and simulated ischemia-reperfusion injury. Stem Cells Dev..

[27] Kim S, Kim C, Park S (2017). Mdivi-1 protects adult rat hippocampal neural stem cells against palmitate-induced oxidative stress and apoptosis. Int. J. Mol. Sci..

[28] Fan SM (2016). Magnetic resonance diffusion tensor imaging study of rhesus optic nerve radiation injury caused by a single dose/fractionation scheme stereotactic radiosurgery at an early stage. J. Neuroradiol..

[29] Liu C (2018). APP upregulation contributes to retinal ganglion cell degeneration via JNK3. Cell Death Differ..

[30] You Y, Klistorner A, Thie J, Graham SL (2011). Latency delay of visual evoked potential is a real measurement of demyelination in a rat model of optic neuritis. Invest. Ophthalmol Vis. Sci..

[31] You Y, Klistorner A, Thie J, Gupta V, Graham S (2012). Axonal loss in a rat model of optic neuritis is closely correlated with visual evoked potential amplitudes using electroencephalogram-based scaling. Invest. Ophthalmol Vis. Sci..

[32] Pont LP (2017). Immediate breast reconstruction with abdominal free flap and adjuvant radiotherapy. Plast. Reconstr. Surg..

[33] Esassolak M (2004). Evaluation of the effects of radiotherapy to the chiasm and optic nerve by visual psychophysical and electrophysiologic tests in nasopharyngeal carcinoma. Int. J. Radiat. Oncol..

[34] Mishra P, Chan DC (2014). Mitochondrial dynamics and inheritance during cell division, development and disease. Nat. Rev. Mol. Cell Biol..

[35] Praefcke GJK, McMahon HT (2004). The dynamin superfamily: universal membrane tubulation and fission molecules?. Nat. Rev. Mol. Cell Biol..

[36] Hoppins S, Lackner L, Nunnari J (2007). The machines that divide and fuse mitochondria. Annu. Rev. Biochem..

[37] Xiao L (2014). Rap1 ameliorates renal tubular injury in diabetic nephropathy. Diabetes.

[38] Sharp WW (2015). Inhibition of the mitochondrial fission protein dynamin-related protein 1 improves survival in a murine cardiac arrest model. Crit. Care Med..

[39] Grohm J (2012). Inhibition of Drp1 provides neuroprotection in vitro and in vivo. Cell Death Differ..

[40] Fan L (2017). Mdivi-1 ameliorates early brain injury after subarachnoid hemorrhage via the suppression of inflammation-related blood–brain barrier disruption and endoplasmic reticulum stress-based apoptosis. Free Radic. Biol. Med..

[41] Ding M (2017). Inhibition of dynamin-related protein 1 protects against myocardial ischemia-reperfusion injury in diabetic mice. Cardiovasc. Diabetol..

[42] Bordt EA (2017). The putative Drp1 inhibitor mdivi-1 is a reversible mitochondrial complex I inhibitor that modulates reactive oxygen species. Dev. Cell.

[43] Lee CA, Chin L, Li L (2018). Hypertonia-linked protein Trak1 functions with mitofusins to promote mitochondrial tethering and fusion. Protein Cell.

[44] Taguchi N, Ishihara N, Jofuku A, Oka T, Mihara K (2007). Mitotic phosphorylation of dynamin-related GTPase Drp1 participates in mitochondrial fission. J. Biol. Chem..

[45] Jahani-Asl A (2015). CDK5 phosphorylates DRP1 and drives mitochondrial defects in NMDA-induced neuronal death. Hum. Mol. Genet..

[46] Liang Z (2015). Cdk5 regulates activity-dependent gene expression and dendrite development. J. Neurosci..

[47] Park J (2015). Iron overload triggers mitochondrial fragmentation via calcineurin-sensitive signals in HT-22 hippocampal neuron cells. Toxicology.

[48] Flippo KH (2018). AKAP1 protects from cerebral ischemic stroke by inhibiting Drp1-dependent mitochondrial fission. J. Neurosci..

[49] Prieto J (2016). Early ERK1/2 activation promotes DRP1-dependent mitochondrial fission necessary for cell reprogramming. Nat. Commun..

[50] Huang E (2010). The role of Cdk5-mediated apurinic/apyrimidinic endonuclease 1 phosphorylation in neuronal death. Nat. Cell Biol..

[51] Shirai K (2006). Differential effects of x-irradiation on immature and mature hippocampal neurons in vitro. Neurosci. Lett..

[52] Parihar VK, Limoli CL (2013). Cranial irradiation compromises neuronal architecture in the hippocampus. Proc. Natl Acad. Sci. USA.

[53] Yamamori T (2012). Ionizing radiation induces mitochondrial reactive oxygen species production accompanied by upregulation of mitochondrial electron transport chain function and mitochondrial content under control of the cell cycle checkpoint. Free Radic. Biol. Med..

