# Supplementary material for: Cdk5-mediated Drp1 phosphorylation drives mitochondrial defects and neuronal apoptosis in radiation-induced optic neuropathy
Source: Cell Death Dis. 2020 Sep 3;11(9):720. doi: 10.1038/s41419-020-02922-y (PMC7473761; doi:10.1038/s41419-020-02922-y)
Supplement: Supplementary file 1 — Supplementary figure legends [file 41419_2020_2922_MOESM1_ESM.docx]

Supplementary Figure legends for

**Cdk5-mediated DRP1 phosphorylation drives mitochondrial defects and neuronal apoptosis in radiation-induced optic neuropathy**

**Figure S1.** Cellular viability at different times (12, 24 or 48 h) after 2 Gy (A) and 10 Gy (B) irradiation. (C) Other mitochondrial fission receptor proteins expression were determined using western blotting, and (D) statistical analysis on expression changes conducted. (E) Confocal low magnification images labeled Tom20. Scale bar is 25 µm. Data are presented as the mean ± SD (n = 3-6), *P < .05; **P < .01; ***P< .001.

**Figure S2****.** (A) Cell viability of 5 µmol Mdivi-1 treated after 12 h irradiation. (B) The specific inhibition of Drp1 was measured by Western blotting with three different siRNA sequences. (C) ROS generation was detected after 6 Gy irradiation with either ncRNA- or siRNA-pre-treatment. Data are presented as the mean ± SD (n = 3-6), *P < .05; **P < .01; ***P< .001.

**Figure S3.** (A) Cellular viability was measured with different concentrations (10, 15 and 20 µmol) of roscovitine incubated after 12 h of 6 Gy irradiation. (B) Cell apoptosis with two inhibitors treated was examined after 24 h irradiation using flow cytometry, and apoptotic cells were calculated. Data are presented as the mean ± SD (n = 3-6), *P < .05; **P < .01; ***P< .001.

**Figure S4.** (A) The specific inhibition of Cdk5 was measured with three different siRNA sequences. (B) After incubated with si-Cdk5 and si-Drp1, cell apoptosis was detected using Annexin-V/PI staining, and (C) the ratio of apoptotic cells was calculated. Data are presented as the mean ± SD (n = 3), *P < .05; **P < .01; ***P< .001.

**Figure S5.** (A) Retinal hematoxylin and eosin staining for 1-3 weeks after 8 Gy and 12 Gy irradiation, and ONL thickness was analyzed at different time points (B). Bar, 100 µm. (C) The method diagram of ONL layer thickness assessment. Bar, 500 µm. (D) TEM image showed localization at RGC layer. Bar, 5 µm. (E) The analysis of mitochondrial perimeter in radiation group at different time points and (F) in inhibitors-treated group. Data are presented as the mean ± SD (n = 3-5), *P < .05; **P < .01; ***P< .001.

**Figure S6.** Levels of Drp1 (Ser616), Drp1 (Ser637), Drp1, Cdk5, and Fis1 were detected in human peripheral blood samples by WB. Samples from two controls, 2 negative controls (NC), and 2 RION patients were used, and quantitative analysis was conducted. Data are presented as the mean ± SD (n = 2), *P < .05; **P < .01; ***P< .001.
