# Supplementary figures and images for: Cdk5-mediated Drp1 phosphorylation drives mitochondrial defects and neuronal apoptosis in radiation-induced optic neuropathy
Source: Cell Death Dis. 2020 Sep 3;11(9):720. doi: 10.1038/s41419-020-02922-y (PMC7473761; doi:10.1038/s41419-020-02922-y)

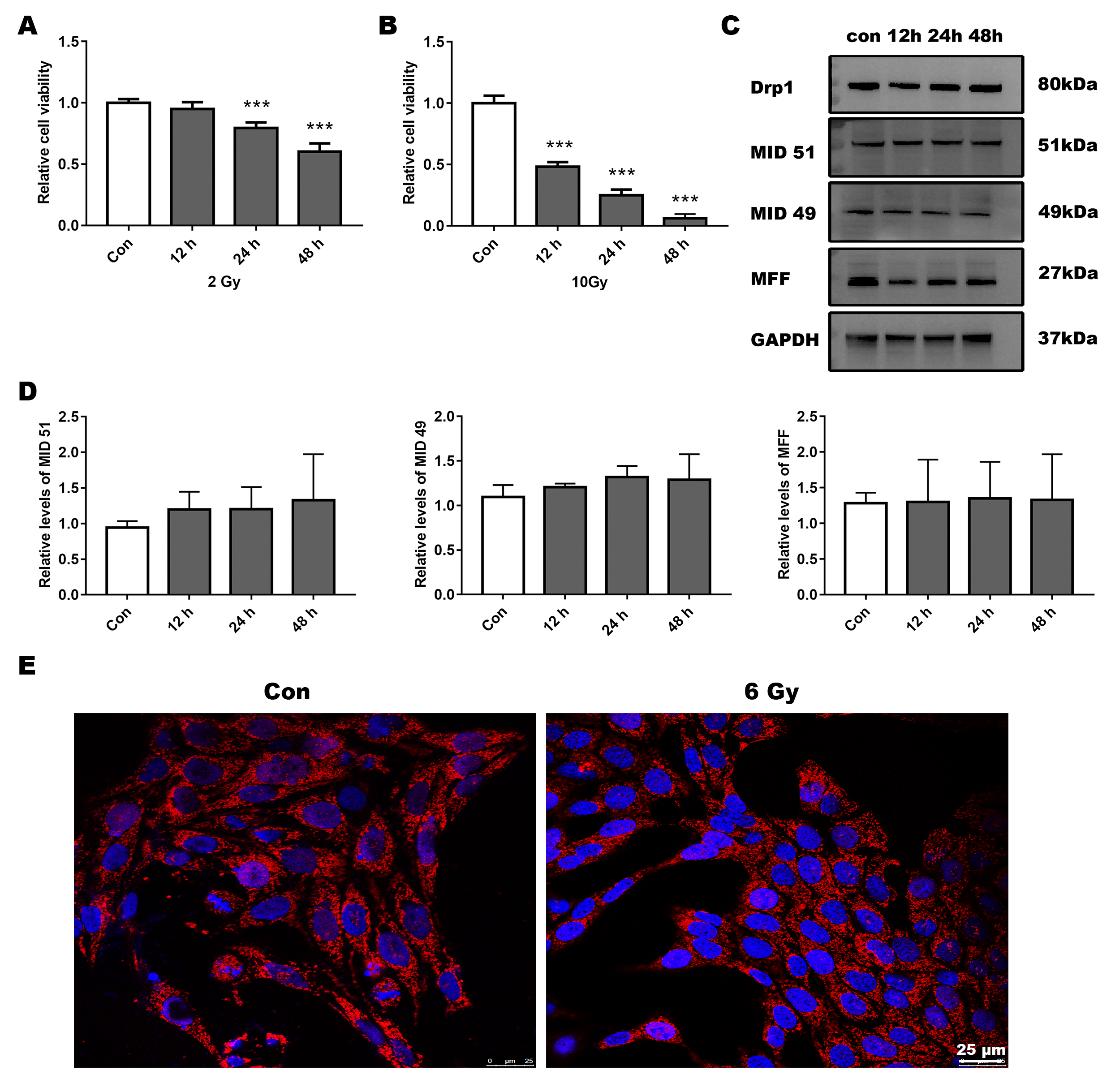

Supplement: Supplementary file 2 — Supplemental Figure 1 [file 41419_2020_2922_MOESM2_ESM.tif]

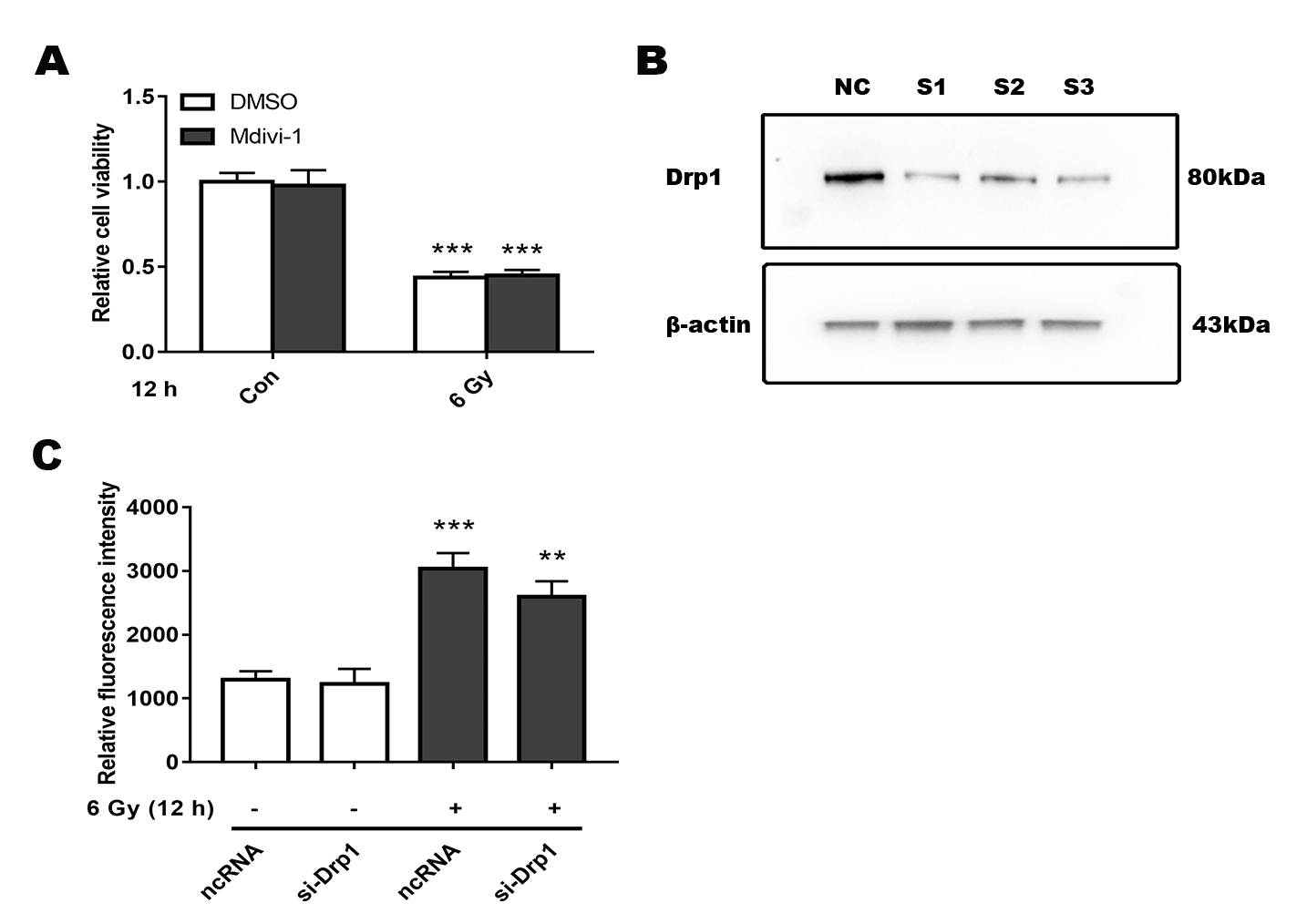

Supplement: Supplementary file 3 — Supplemental Figure 2 [file 41419_2020_2922_MOESM3_ESM.tif]

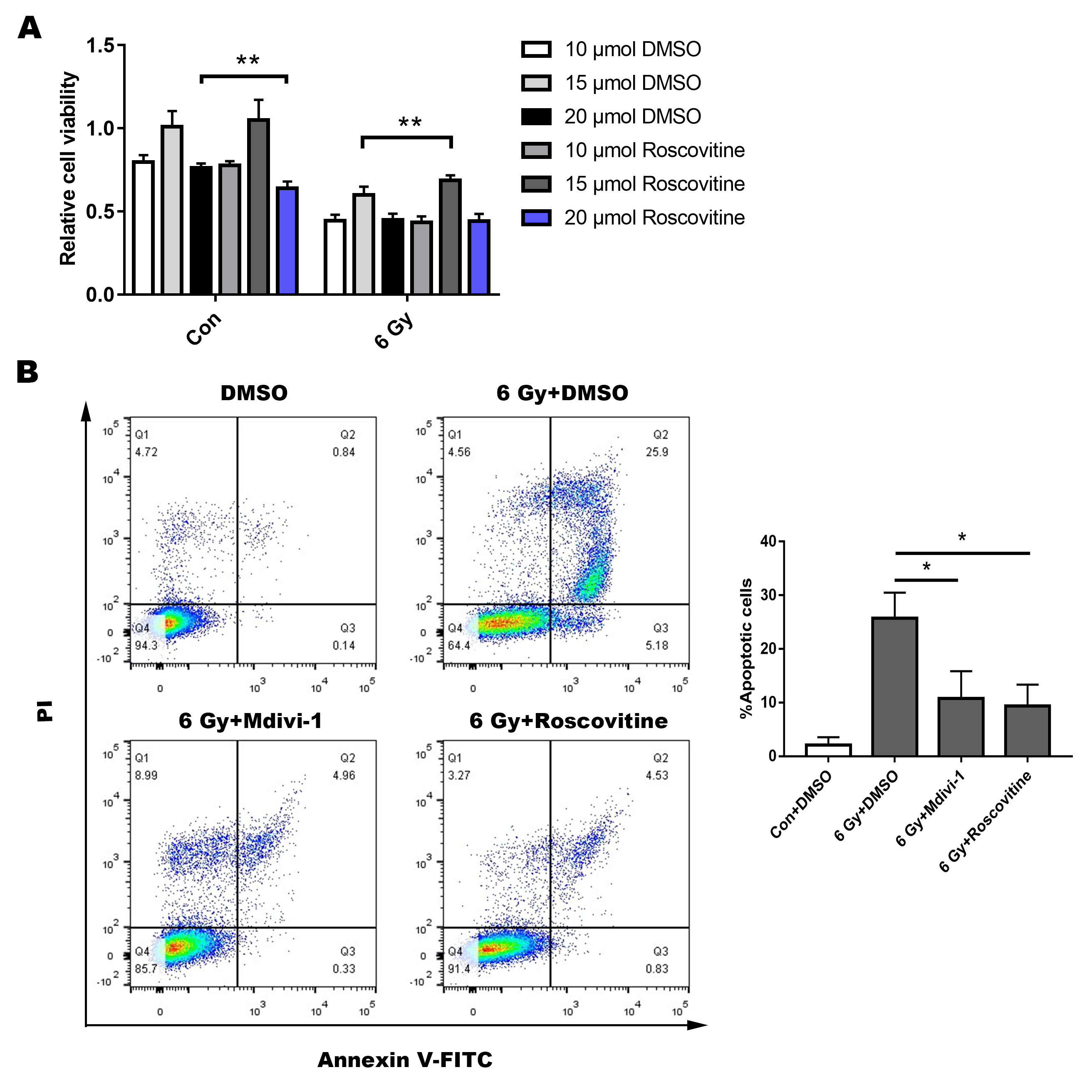

Supplement: Supplementary file 4 — Supplemental Figure 3 [file 41419_2020_2922_MOESM4_ESM.tif]

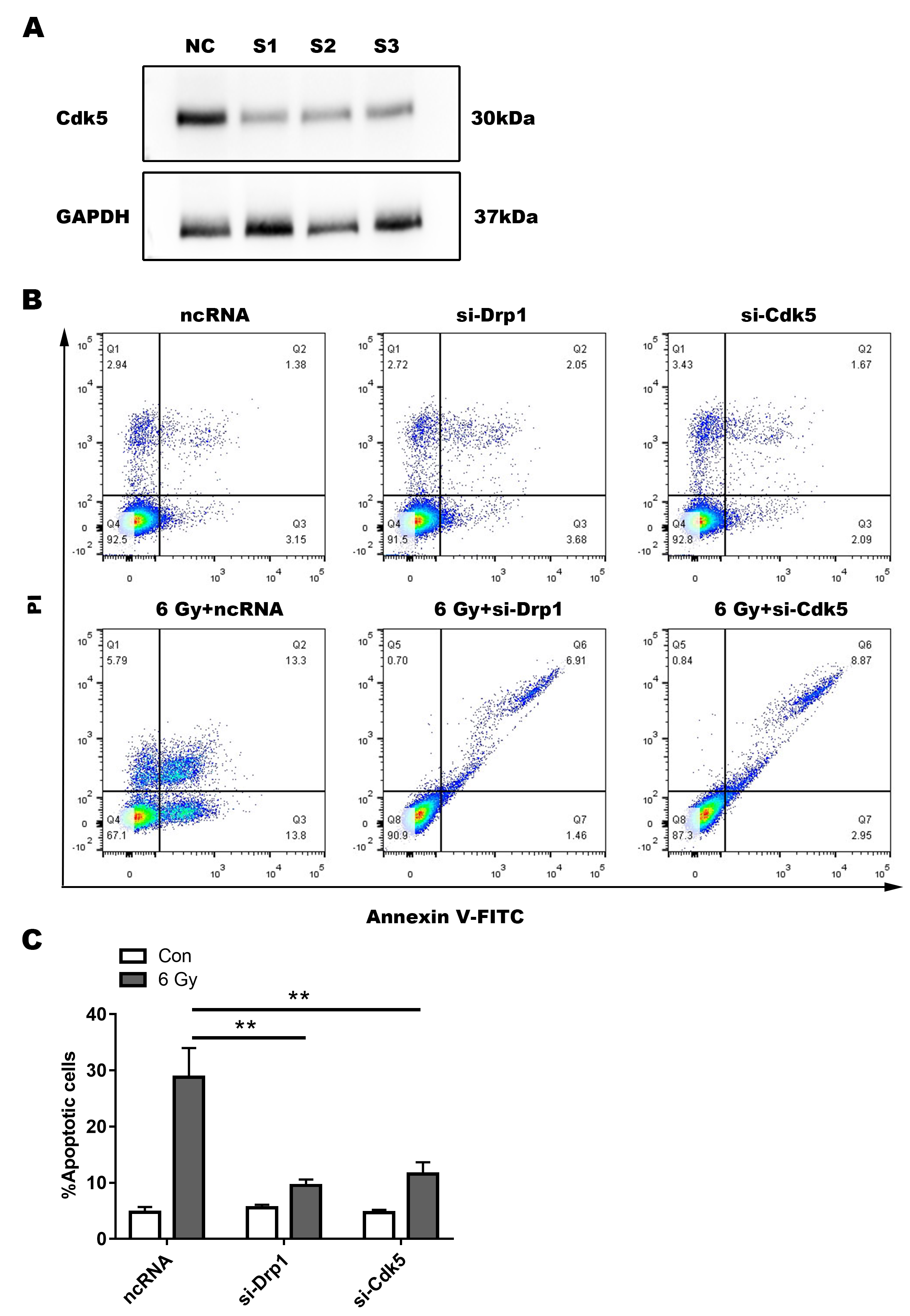

Supplement: Supplementary file 5 — Supplemental Figure 4 [file 41419_2020_2922_MOESM5_ESM.tif]

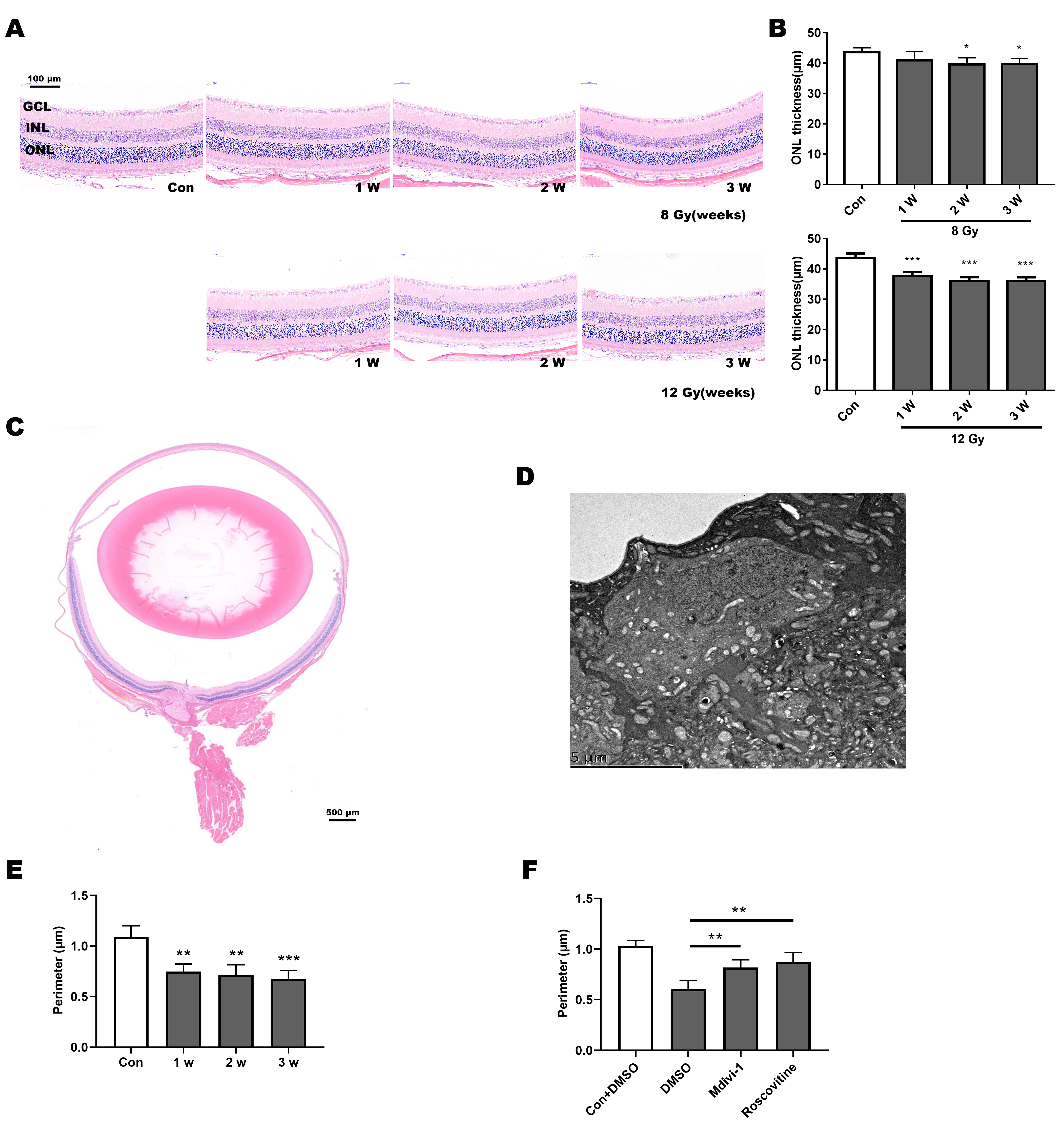

Supplement: Supplementary file 6 — Supplemental Figure 5 [file 41419_2020_2922_MOESM6_ESM.tif]

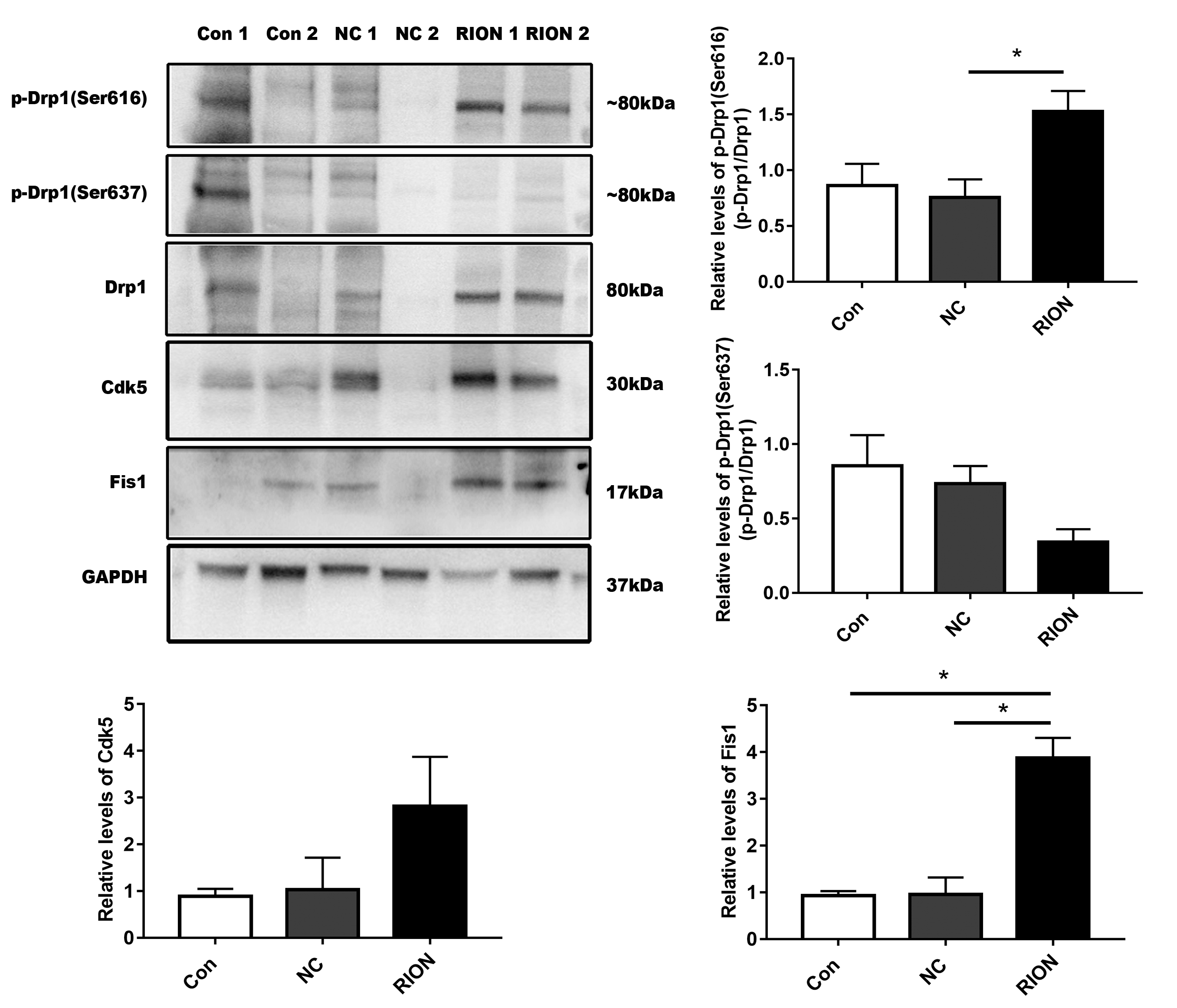

Supplement: Supplementary file 7 — Supplemental Figure 6 [file 41419_2020_2922_MOESM7_ESM.tif]
